# Electrical Phase Control Based on Graphene Surface Plasmon Polaritons in Mid-infrared

**DOI:** 10.3390/nano10030576

**Published:** 2020-03-22

**Authors:** Yindi Wang, Hongxia Liu, Shulong Wang, Ming Cai, Haifeng Zhang, Yanbin Qiao

**Affiliations:** 1 Key Laboratory for Wide Band Gap Semiconductor Materials and Devices of Education Ministry, School of Microelectronics, Xidian University, Xi’an 710071, China; wangyindi4213@126.com (Y.W.); cm9999787@163.com (M.C.); 2 Beijing Smart-Chip Microelectronics Technology Co., Ltd. Beijing 100192, China; zzflys@163.com (H.Z.); wyd4213@163.com (Y.Q.)

**Keywords:** grapheme surface plasmon polaritons, phase modulation, Mid-infrared

## Abstract

Phase modulation of light is the core of many optoelectronic applications, such as electro-optic switch, sensors and modulators. Graphene Surface plasmon polaritons (SPPs) exhibit unique properties in phase modulation including dynamic tunability, a small driving voltage and small device size. In this paper, the novel phase modulation capability of graphene SPPs in mid-infrared are confirmed through theory and simulation. The results show that graphene SPPs can realize continuous tuning of the phase shift at multiple wavelengths in mid-infrared, covering the phase range from 0° to 360°. Based on these results, a sandwich waveguide structure of dielectric–graphene–dielectric with a device length of 800 nm is proposed, which shows up to 381° phase modulation range at an operating wavelength of 6.55 µm, given a 1 V driving voltage. In addition, the structure size is much shorter than the wavelength in mid-infrared and can realize sub-wavelength operation. This work paves the way to develop graphene-based tunable devices for mid-infrared wave-front control.

## 1. Introduction

Surface plasmon polaritons (SPPs), the electromagnetic waves coupled to charge excitations on the surface of metal, are widely applied in sub-wavelength-scale optical processing [1]. Because of the breakthrough of the diffraction limit and the ultra-compact mode confinement, SPPs have become the cornerstones of various applications, including optical metamaterials, ultra-sensitive optical biosensors [2,3,4] and quantum information processing [5]. In recent years, SPPs based on metals have been widely used, because most studies on SPPs focus on precious metals represented by silver and gold [4,6,7,8,9]. However, even noble metals, which are widely regarded as the best available SPPs materials, are hardly tunable. They also suffer large decoherence and exhibit large ohmic losses, which limit their application in light manipulation equipment. In this context, it is necessary to search for more excellent SPP materials to realize high tunability, lower transmission loss and stronger constraint [10].

Graphene, a monolayer of carbon atoms arranged in a honeycomb lattice [11], has been widely used in optical devices because of its excellent electro-optical performance [12,13,14]. Previous research on graphene has proven that this material is a zero-gap semiconductor and its chemical potential can be tuned dynamically [15], which can be doped to high values of carrier concentrations either electrically or chemically. These unique properties make graphene a potential material for tunable optical devices such as modulators, polarizers, sensors, etc. Due to their tunability and small size, these graphene-based devices have advantages over traditional devices. In addition, graphene has been proven to support SPPs from a mid-infrared band to THz and SPPs bound to the surface of doped graphene, which exhibit a number of favorable properties [16,17]. The ability to fabricate large-sized, high-crystalline samples enables the lifetime of SPPs to reach hundreds of optical cycles, making graphene a potential alternative for precious metal SPPs [12].

The phase modulation of light is at the core of many applications and much research [18]. Phase modulation in mid-infrared can be applied to the design of a phased array radar, atmospheric communication equipment, mid-infrared detector, etc. [19,20]. It is a huge challenge to accomplish the design of these devices efficiently with a small device size [21]. However, traditional phase modulators in mid-infrared are mainly based on waveguides employing electro-optic materials such as GaAs and LiNbO_3_, which have large device sizes and require high external driving voltages [22,23]. Besides, these phase modulators suffer relatively large power dissipation and weak tunability, which greatly limits their application in light manipulation equipment. In this context, graphene SPPs shows great potential in mid-infrared phase modulation, which exhibits unique properties as a phase modulation platform with dynamic tunability, small footprint and small drive voltage [21]. The carrier concentration of graphene can be tuned over a wide range by electrostatic gate [24,25]. However, at present, the study of mid-infrared phase modulation based on graphene is mostly confined to the regulation function of graphene, and the application of graphene SPPs in mid-infrared phase modulation has not been studied systematically. This paper theoretically demonstrates the phase modulation of mid-infrared light using electrically tunable graphene SPPs.

In this paper, we explore graphene SPPs phase modulation in mid-infrared by presenting a sandwich waveguide structure of dielectric–graphene–dielectric, which can realize phase modulation from 0° to 360° in situ. The structure length of 800 nm is much shorter than the free-space wavelength in mid-infrared, realizing sub-wavelength operation. Here, we provide a scattering theory for graphene SPPs’ propagating and phase modulation through theoretical derivation and software simulation. The modulation is achieved by controlling the voltage applied to the graphene where the spatial carrier density is tunable. This work constitutes a first step for the application of graphene phase modulation in photoelectric devices such as ultracompact modulators and sensors.

## 2. Structure Design

Previous structural designs have employed metals directly patterned to graphene as gate electrodes [26,27,28]. Contact between graphene and metal has been proved to cause a Fermi level pinning effect [29,30]. This effect can severely restrict the modification range of graphene Fermi energy level, which weakens the phase modulation ability of graphene [31]. The graphene-based phase modulation structure proposed here avoids these deleterious effects. In addition, large contact resistance between the metal and graphene can also be avoided.

**Figure 1:**
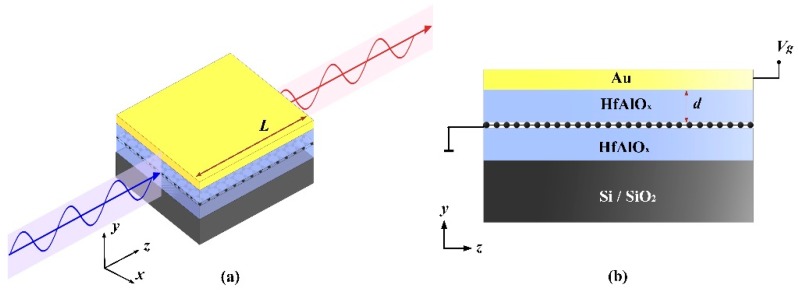
Schematic illustration of the proposed structure: (**a**) 3D layout; (**b**) cross-section.

HfAlO_x_ has the advantages of a low charge trap density and high mobility. It can contact graphene perfectly without destroying the carrier mobility of grapheme. As shown in Figure 1, the presented structure consists of a silicon or silicon-dioxide (SiO_2_) substrate [32], 100 nm HfAlO*_x_* isolation layer, monolayer graphene, HfAlO*_x_* dielectric layer with a thickness of *d*, and a 50 nm thick gold electrode. Here, Chemical Vapor Deposition (CVD) is used to grow monolayer grapheme, and then can be transferred to HfAlO*_x_*. As revealed in Figure 1a, the length of the structure is *L*, which is also the length of the optical path. In this structure, the HfAlO*_x_* dielectric layer serves as an effective high-k dielectric for graphene chemical potential tuning when external voltage (*V_g_*) is applied between graphene and the metal gate. Here, the 100 nm HfAlO*_x_* isolation layer isolates graphene from the environment, which is conducive to maintaining the long-term stability of graphene and preventing the accumulation of substances adsorbed on graphene [30,33,34,35].

## 3. Methods

Because SPPs are excited at the appropriate chemical potential, graphene has a strong response to mid-infrared electromagnetic waves [36]. In addition, the carrier concentration of graphene can be easily adjusted by electrostatic gating due to its atomic thickness and the linear density of electronic states [12]. For this proposed structure, the charge carrier density can be calculated with a parallel plate capacitor model [37]. The dielectric constant of graphene can be adjusted in a wide range [21]. The electrical phase control of graphene in mid-infrared band mainly depends on the electrical tunable carrier concentration and chemical potential of graphene.

Graphene can be considered as a two-dimensional material, and its surface conductivity *σ* is related to the radiation angular frequency *ω*, chemical potential *μ*, and relaxation time. The conductivity of monolayer graphene can be obtained from Kubo formula [10]

(1) $$\sigma_{\mathrm{int}ra}=\sigma_{0}\frac{4\mu }{\pi }\frac{1}{\hbar \tau_{1}-i\hbar \omega }$$

(2) $$\sigma_{\mathrm{int}\mathrm{er}}^{\prime }=\sigma_{0}(1+\frac{1}{\pi }\arctan\frac{\hbar \omega -2\mu }{\hbar \tau_{2}}-\frac{1}{\pi }\arctan\frac{\hbar \omega +2\mu }{\hbar \tau_{2}})$$

(3) $$\sigma_{\mathrm{int}er}^{\prime \prime }=-\sigma_{0}\frac{1}{2\pi }\ln\frac{{\left(2\mu +\hbar \omega \right)}^{2}+\hbar^{2}\tau_{2}^{2}}{{\left(2\mu -\hbar \omega \right)}^{2}+\hbar^{2}\tau_{2}^{2}}$$

(4) $$\sigma =\sigma_{\mathrm{int}ra}+\sigma_{\mathrm{int}er}^{\prime }+i\sigma_{\mathrm{int}er}^{\prime \prime }$$

where *τ_1_* = 10 fs is the in-band relaxation time of graphene, and *τ_2_* = 1.2 ps is the inter-band relaxation time of graphene. *σ_0_ = πe^2^/2h*; *ħ* = 1.055 × 10^−34^ J·s is the reduced Planck’s constant.

**Figure 2:**
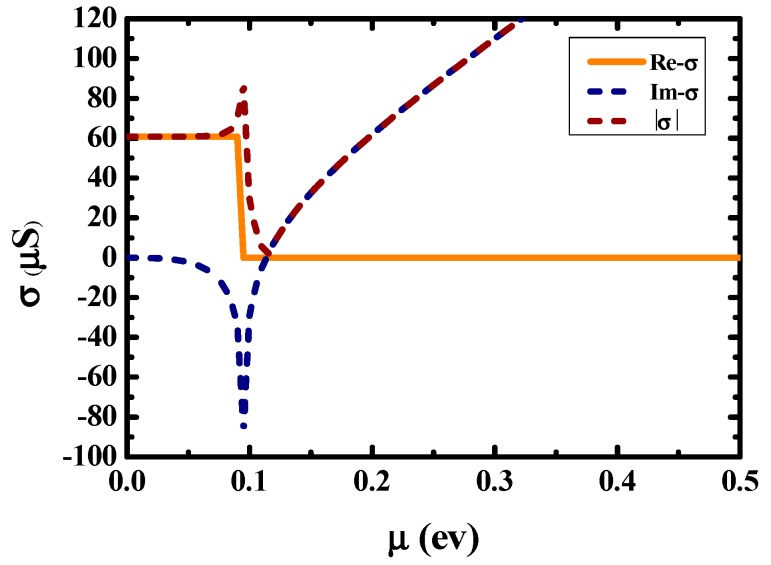
Dependence of conductivity on chemical potential.

By solving simultaneous Equations (1)–(4), the dependence of electrical conductivity on chemical potential of graphene will be obtained. Given a working wavelength 6550 nm, the relationship between graphene electrical conductivity and chemical potential is shown in Figure 2.

The chemical potential of graphene can be tuned by external applied voltage [10]. The chemical potential of the graphene layer can be determined by the carrier concentration *n_0_*

(5) $$\mu =\hbar v_{f}\sqrt{\pi \cdot n_{0}}=\hbar v_{f}\sqrt{\pi \frac{\varepsilon_{0}\varepsilon_{r}}{d\cdot e}(V_{g}+v_{0})}$$

where *n_0_ = ε_0·_ε_r_(v+v_0_)/(d*·*e)*. *v_f_* = 1.1×10^6^ m/s is the Fermi velocity. *v_0_* is the offset voltage caused by natural doping, and its value is 0. *ε_0_* and *ε_r_* are dielectric constant in free space and relative dielectric constant of the substrate material, respectively. *d* is the thickness of graphene substrate (the dielectric between gate and graphene), and *V_g_* is the voltage applied to graphene.

Graphene in the proposed sandwich structure can be regarded as a layer of the current. By solving Maxwell equations and the boundary conditions of Maxwell equations, we can obtain the dispersion relation of SPPs wave in TM mode as shown in the following Equation (6) [10]

(6) $$\frac{\varepsilon_{1}}{k_{1}}+\frac{\varepsilon_{2}}{k_{2}}+\frac{i\sigma }{\omega \varepsilon_{0}}=0$$

where *k_1_* and *k_2_* are P-wave vectors of the SPPs wave in upper and lower dielectric layers, respectively. The relationship between P-wave vectors and the propagation constant of SPPs (*β*) can be expressed as: *k_m_^2^ = β^2^-ε_m_k_0_^2^*, where *m* = 1, 2. In the proposed structure, *ε_1_ = ε_2_ = ε_r_* (relative dielectric constant of HfAlO*_x_*), and we can get the dispersion relation of graphene, as shown in Equation (7) [38]

(7) $$\beta =k_{0}\sqrt{\varepsilon_{r}-{\left(\frac{2\varepsilon_{r}}{\eta_{0}\sigma }\right)}^{2}}$$

where *k_0_* is the propagation constant of electromagnetic waves in air. *η_0_* = 377 ohms is the impedance of free space.

Since the attenuation length of SPPs wave is much shorter than the thickness of the HfAlO*_x_* medium, it is not necessary to consider the spatial structure of the metal gate. From TM mode wave equation *H_x_(y) = A_m_e^iβz^e^-kmy^,* it can be seen that different phases *ϕ* are accumulated after propagation through different path lengths. The phase shifts *Δϕ* in different optical transmission lengths can be calculated by optical path length *L* and the real part of *β*, which can be expressed as

(8) Δϕ=Re(β)⋅L

It can be obtained from the above equations that the propagation constant in transmission direction (*β*) can be tuned by different voltages applied to graphene and different thicknesses of HfAlO*_x_*. Thus, the phase shift of the graphene SPPs wave can be adjusted. The graphene SPPs phase shift is tunable in situ, and can be varied spatially, making it a unique platform for transformation optics in two dimensions

## 4. Results and Discussion

Through the discussions in the method section, we can conclude that the graphene SPPs phase shift is affected by a series of factors, including applied voltage (*V_g_*), HfAlO*_x_* dielectric thickness (*d*), incident wavelength, the dielectric constant of the HfAlO*_x_* (*ε_r_*) and optical path length (*L*). To further research the influence of all the factors on phase shift, the classical control variable method is adopted here. *L* is fixed at 800 nm in this structure, which can guarantee a completely adjustable phase shift from 0° to 360° in a voltage range of 5 V. In the simulations, the relative permittivities of Si and SiO_2_ are 11.9 and 2.09, respectively [39,40,41,42]. 

**Figure 3:**
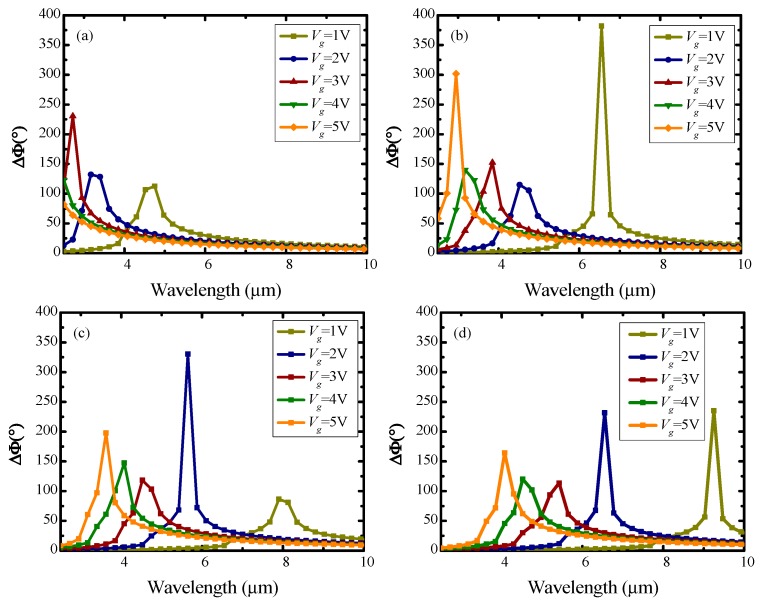
Dependence of phase shift on applied voltage and incident wavelengths at different HfAlO*_x_* dielectric thickness: (**a**) *d* = 50 nm; (**b**) *d* = 100 nm; (**c**) *d* = 150 nm; (**d**) *d* = 200 nm.

The plots of the simulated phase shift (*Δϕ*) on incident wavelengths for different HfAlO*_x_* dielectric thickness (50, 100, 150, 200 nm) are shown in Figure 3a–d, respectively, incident wavelength is ranged from 2500 to 25,000 nm (mid-infrared region), and the dielectric constant of the HfAlO*_x_* used in this simulation is set at 14.3, which is in common use. As revealed in Figure 3, phase shift is selective to wavelength, and the maximum phase shift point shows blue-shift with increasing applied voltage, which is mainly because the tuned graphene conductivity resulted from the different applied voltage. The maximum phase shift shows nonlinear changes with different voltages. This depends on the nonlinear relationship between voltage and conductivity, conductivity and propagation constant. As revealed from the simulation results, phase shift of 360° can be reached at all wavelengths in mid-infrared band, by adjusting the applied voltage, substrate thickness and optical path length.

**Figure 4:**
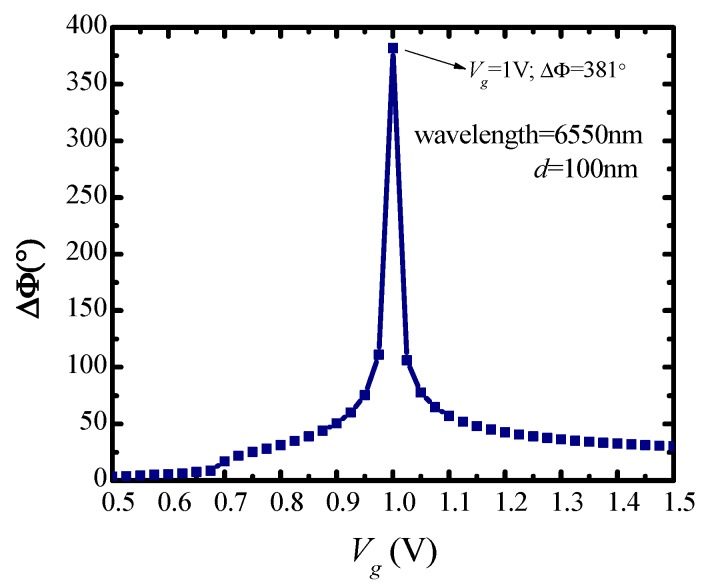
Dependence of phase shift on applied voltage. Incident wavelength is 6550 nm, dielectric material is HfAlO*_x_* and *d* = 100 nm.

It can be obtained from Figure 3b that the phase shift reaches 381° at 6550 nm when *d* = 100 nm, *V_g_* = 1 V. Next, fixing incident wavelength at 6550 nm, the influence of continuous applied voltage variation on phase shift is researched, as shown in Figure 4. As can be seen from Figure 4, the phase shift peak (381°) appears at 1 V during the voltage variation, with a dielectric thickness of 100 nm. In this case, given a voltage ranging from 0 to 1 V applied to graphene, a phase modulation of 0° to 381° can be obtained.

**Figure 5:**
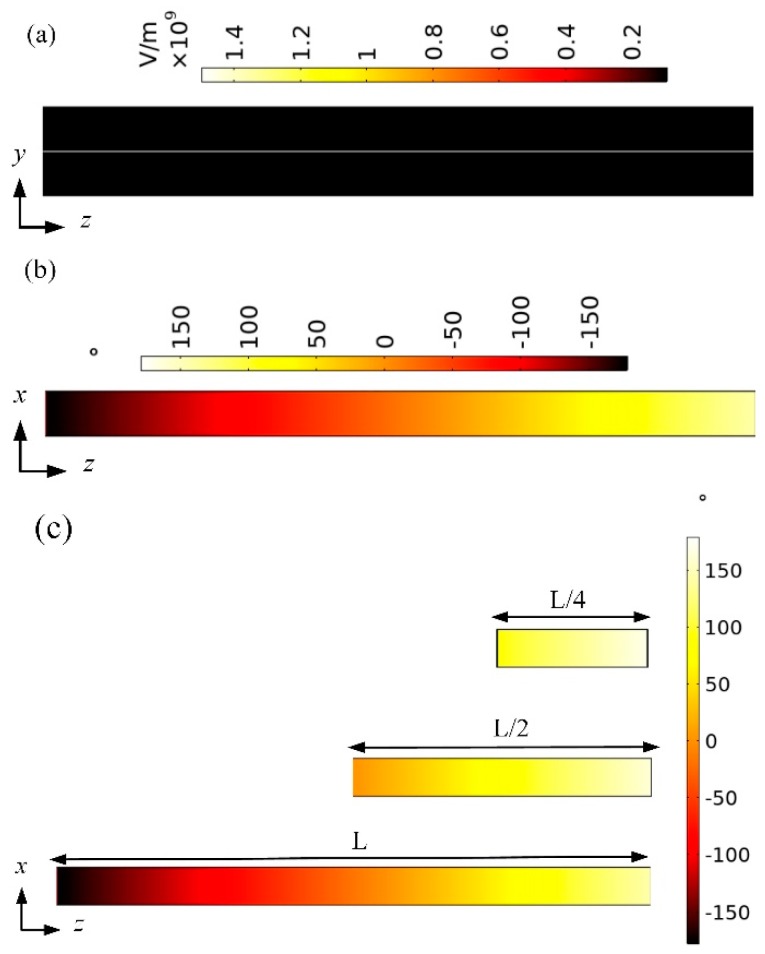
Simulation results of the proposed structure: (**a**) electric field distributions of SPPs guided by the proposed structure; (**b**) phase shift in the SPPs wave in the direction of transmission; (**c**) phase shift comparison of different transmission lengths.

To further confirm the theoretical results presented above, the phase modulation performance of the presented structure is simulated and verified using an RF module of COMSOL Multiphysics software. Here, the operating wavelength is 6550 nm with *V_g_* fixed at 1 V and *d* fixed at 100 nm. The electric field distribution of the SPPs wave guided by the presented structure is shown in Figure 5a. It can be clearly observed that the SPPs wave is excited on the surface of graphene and dielectric medium. Figure 5b shows the phase shift in the SPPs wave in the direction of transmission. The phase shift comparison under different transmission lengths (*L*) is shown in Figure 5c. It can be concluded from the simulation results that the structure achieves 360-degree phase shift, which is consistent with the theoretical results.

**Figure 6:**
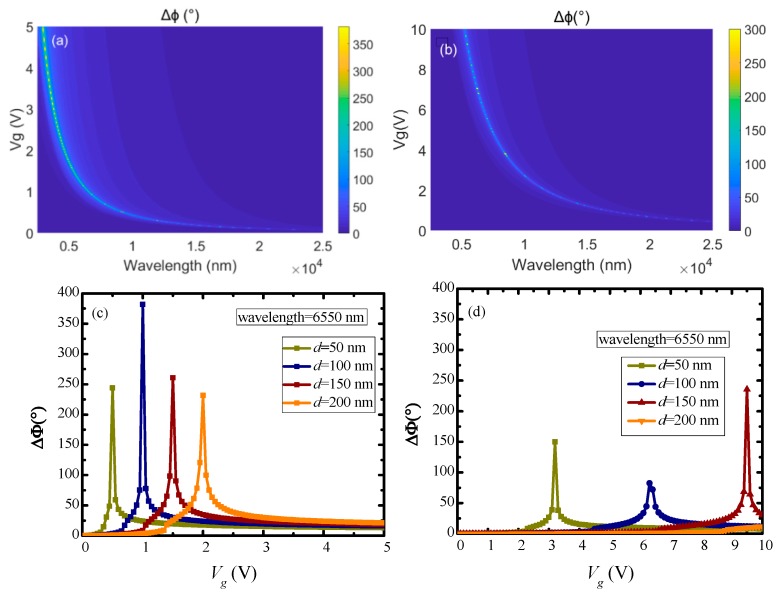
Comparison of phase modulation properties for different dielectric materials: (**a**) simulated tunable phase shift of mid-infrared with voltage variation for HfAlO*_x_* dielectric; (**b**) simulated tunable phase shift of mid-infrared with voltage variation for SiO_2_ dielectric; (**c**) dependence of phase shift on applied voltage at 6550 nm for HfAlO*_x_* dielectric; (**d**) dependence of phase shift on applied voltage at 6550 nm for Si dielectric.

In addition, the simulation comparison between HfAlO*_x_* and SiO_2_ was carried out to demonstrate the superiority of HfAlO*_x_* as a dielectric material. Figure 6a,b shows the simulated tunable phase shift of mid-infrared with voltage variation. Figure 6c,d shows the dependence of phase shift on applied voltage at 6550 nm wavelength for different dielectric layer thicknesses. As revealed in Figure 6c,d, the depth of phase modulation and the best modulation voltage point are changed with different dielectric layer thicknesses (*d*). It can be observed clearly that HfAlO*_x_* has the obvious advantages of a low operating voltage compared with SiO_2_. Besides this, excellent phase modulation property and perfect contact with graphene make HfAlO*_x_* the best choice for dielectric material [35].

**Figure 7:**
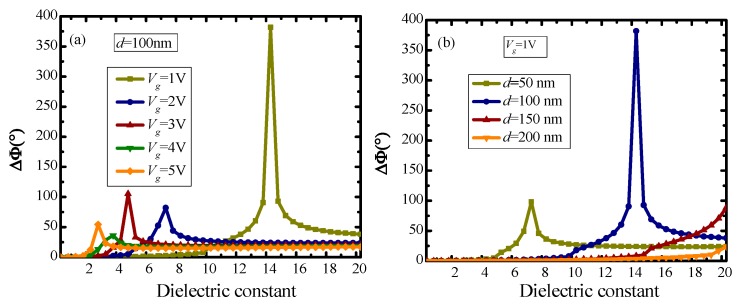
Dependence of the dielectric constant of HfAlO*_x_* on the phase shift: (**a**) given different *v_g_*; (**b**) given different *d*.

The dielectric constant of HfAlO*_x_* used in the previous simulations is 14.3. In order to prove that 14.3 is the optimal dielectric constant of HfAlO*_x_* under the design parameters of the presented structure, the influence of the dielectric constant of HfAlO*_x_* on the phase shift characteristics of the presented structure was discussed here. The value of the dielectric constant of HfAlO*_x_* is about between 9 and 20, which is dependent on the manufacturing process and the stoichiometric ratio of Hf, Al and O. Which dielectric constant is the optimal constant depends on other parameters, including applied voltage (*V_g_*) and HfAlO*_x_* dielectric thickness (*d*), and the relationship between them is expressed by the formulas shown in the methods. In this simulation, we set the incident wavelength at 6550 nm. As revealed in Figure 7, phase shift is selective to the dielectric constant of HfAlO*_x_*, and the dielectric constant of HfAlO*_x_* at the maximum phase shift point is different when given different *V_g_* and *d*. This is mainly because the different graphene conductivity resulted from the different *V_g_* and *d*. The maximum of phase shift shows nonlinear changes, which depends on the nonlinear relationship between *V_g_*, *ε_r_* and *d*. As revealed from Figure 7, 14.3 is the optimal dielectric constant of HfAlO*_x_* for the presented structure.

## 5. Conclusions

In this paper, we investigate the phase modulation properties of graphene SPPs in detail. To numerically demonstrate the feasibility of graphene SPPs’ phase modulation, a sandwich waveguide structure of dielectric–graphene–dielectric is presented here. It is found that this structure can realize the continuous tuning of the phase shift in mid-infrared, covering the phase range from 0° to 360°. Given HfAlO*_x_*, the dielectric thickness of 100 nm and drive voltage of 1 V, the proposed structure can realize a phase shift of 381° at 6.55 um, where only an 800 nm optical path is needed. The results in this paper confirm the unique properties of graphene SPPs in phase modulation and demonstrate the great potential of graphene SPPs phase modulation in mid-infrared, which can be widely applied to the design of on-chip interferometers and tunable modulators.
